# Differences According to Age in the Diagnostic Performance of Cardiac Biomarkers to Predict Frailty in Patients with Acute Heart Failure

**DOI:** 10.3390/biom12020245

**Published:** 2022-02-02

**Authors:** Lara Aguilar-Iglesias, Ana Merino-Merino, Ester Sanchez-Corral, Maria-Jesus Garcia-Sanchez, Isabel Santos-Sanchez, Ruth Saez-Maleta, Jose-Angel Perez-Rivera

**Affiliations:** 1 Department of Cardiology, University Hospital of Burgos, 09006 Burgos, Spain; lara.aguigle@hotmail.com (L.A.-I.); anamerinomerino@gmail.com (A.M.-M.); corral.esther@gmail.com (E.S.-C.); chusagarcia@hotmail.com (M.-J.G.-S.); aiss.isabelsantos@gmail.com (I.S.-S.); 2 Department Clinical Analysis, University Hospital of Burgos, 09006 Burgos, Spain; rsaezu@saludcastillayleon.es; 3 Facultad de Ciencias de la Salud, Universidad Isabel I, 09003 Burgos, Spain

**Keywords:** biomarkers, heart failure, frailty, elderly, NT-proBNP

## Abstract

Frailty has traditionally been studied in the elderly population but scarcely in younger individuals. The objective of the present study is to analyze differences according to age in the diagnostic performance of cardiac biomarkers to predict frailty in patients admitted to the hospital for acute heart failure (AHF). A frailty assessment was performed with the SPPB and FRAIL scales (score > 3). We included 201 patients who were divided according to age: those older and younger than 75 years. In the younger group, no biomarker was related to the presence of frailty. This was mainly determined by age and comorbidities. In the elderly group, NT-proBNP was significantly related to the presence of frailty, but none of the baseline characteristics were. The best cut-off point in the elderly group for NT-proBNP was 4000 pg/mL. The area under the curve (AUC) for proBNP for frailty detection was 0.62 in the elderly. Another similar frailty scale, the SPPB, also showed a similar AUC in this group; however, adding the NT-proBNP (one point if NT-proBNP < 4000 pg/mL), it showed a slightly higher yield (AUC 0.65). The addition of biomarkers could improve frailty detection in members of the elderly population who are admitted to the hospital for AHF.

## 1. Introduction

Heart failure (HF) is a growing global health problem and has high incidence and prevalence (1–2% of adults) [1]. The prevalence of HF increases with age, with a prevalence of over 70% in those aged 70 years old or older [2]. In patients with HF, frailty is very common. This condition makes patients more vulnerable to the effects of stressors, independent of age [3], and this usually impacts their management and prognosis. In fact, frailty is associated with a higher risk of hospitalization and a higher risk of death [4]. Therefore, frailty assessment is essential in HF patients.

Frailty is usually focused on physical function, such as in the Fried criteria [5], and on clinical variables, such as in the Rockwood criteria [6], which defines frailty as the result of health deficits. According to these criteria, many scales have been developed to help define frailty, such as the FRAIL scale [7] or the SPPB scale [8], which use clinical and functional variables. However, frailty scales do not consider parameters related to the severity of the underlying disease, such as biomarkers. In fact, it is known that high levels of certain biomarkers, such as ST2 or NT-proBNP, involve a worse prognosis in comorbid frail elderly populations with HF [9]. Other biomarkers, such as Galectin-3, are associated with frailty in elderly patients [10]. Therefore, it would be interesting to evaluate the relation between traditional frailty scales and new biomarkers. Moreover, frailty has traditionally been studied in the elderly population, with this assessment being less frequent in younger individuals.

The purpose of this study was to determine differences in the diagnostic performance of cardiac biomarkers according to age to predict frailty in patients admitted to the hospital for acute HF (AHF).

## 2. Materials and Methods

### 2.1. Design and Study Population

We performed a single-center, observational, and cross-sectional study in the University Hospital of Burgos, Spain. We consecutively enrolled all patients with AHF who were admitted to the Cardiology unit of our hospital from July 2020 through May 2021, regardless of age (≥18 years old). AHF was defined according to the European Society of Cardiology guidelines criteria [2]. We excluded those patients with a diagnosis of acute coronary syndrome or stroke in the last 7 days, advanced atrioventricular block at the moment of admission, and systemic disease with a life expectancy of less than 1 year. All of the patients signed written informed consent to participate in the study, and Local Ethics Committee approval was obtained.

### 2.2. Data Collection and Geriatric Assessment

Data were collected by local investigators during the admission process. Baseline clinical features and echocardiographic and ECG data were studied. A blood sample was collected during the first day of admission, and certain prognostic biomarkers were determined: NT-proBNP, ultrasensitive troponin T, c-reactive protein (CRP), hemoglobin, and estimated glomerural filtration rate (GFR). A geriatric assessment was performed on the last day before discharge using the SPPB and FRAIL scales (Supplementary Figures S1 and S2). We used the FRAIL scale to divide the sample into frail patients (FRAIL score > 3) and non-frail patients.

### 2.3. Statistical Analysis

We divided the sample into two groups by age according to a cut-off point of 75 years old. We studied the differences in the biomarkers between the frail and non-frail patients in the two age groups using a *t*-student test. A ROC curve was used to detect the best cut-off point for the biomarkers that showed statistical significance for frailty detection. Finally, we compared the diagnostic performance, which was measured by the area under the curve (AUC), of the SPPB scale to detect frailty in each age group. The results were presented as a number (percentage) for the discrete variables and mean ± standard deviation for the continuous variables. Significant results were deemed statically significant if the *p*-value < 0.05. Statistical analysis was performed using SPSS Statistics for Windows software, version 20.0 (IBM, Chicago, IL, USA).

## 3. Results

### 3.1. Sample Description

We included 201 patients with a mean age of 73 ± 12 years. Of them, 78 (38.6%) were women. In the whole population, 68 (33.7%) of the patients presented frailty defined by a FRAIL score > 3, and the mean FRAIL score was 1.88 ± 1.48. Comorbidities were frequent in our sample, especially hypertension, which was detected in 140 (69.3%) patients, and diabetes, which was detected in 64 (31.7%) patients. There were 30 (14.9%) patients with GFR < 60 mL/min/1.73 m*^2^* and 22 (10.9%) patients with chronic obstructive pulmonary disease. According to the type of HF, 89 (44.1%) patients had HF with a preserved ejection fraction, 27 (13.7%) had HF with a mildly reduced ejection fraction, and 86 (42.6%) had HF with a reduced ejection fraction. HF therapy prescribed before admission is shown in Supplementary Table S1.

The sample comprised 99 patients (49%) who were older than 75 years old and 102 patients who were younger than 75 years old. Frailty according to the FRAIL score criteria was more common in patients over 75 years old than it was in younger patients (2.43 ± 1.48 vs. 1.34 ± 1.28; *p* < 0.001).

### 3.2. Analysis of Frailty Determinants in Each Group

In the group of young patients, NT-proBNP, ST2, and CRP were numerically higher in frailty patients, but no biomarker was significantly related to frailty (Table 1). Furthermore, the frailty patients presented a lower value of hemoglobin and worse GFR, no significant differences were observed.

In this group, frailty was mostly determined by clinical variables (age, hypertension, and diabetes). Frail patients were older (68 ± 5 vs. 62 ± 9 years old), and presented with hypertension more frequently (14 (82.5%) patients vs. 48 (56.5%) patients; *p* = 0.046) and diabetes (10 (58.8%) patients vs. 21 (24.7%) patients; *p* = 0.005).

In the group of patients older than 75 years old, only NT-proBNP was related to frailty (12,297.61 ± 13,710.20 pg/mL vs. 7709.40 ± 8374.26 pg/mL; *p* = 0.046) (Table 2). The rest of the biomarkers presented a non-significant increase in frail patients. No clinical baseline variable had a significant effect on frailty in this group, but frail patients had non-significantly more comorbidities. The best cut-off point for detecting frailty using proBNP was 4000 pg/mL (sensitivity: 78.4%; specificity: 45.8%). The AUC for NT-proBNP was 0.62.

### 3.3. Inclusion of Biomarkers in SPPB Scale

**Figure 1:**
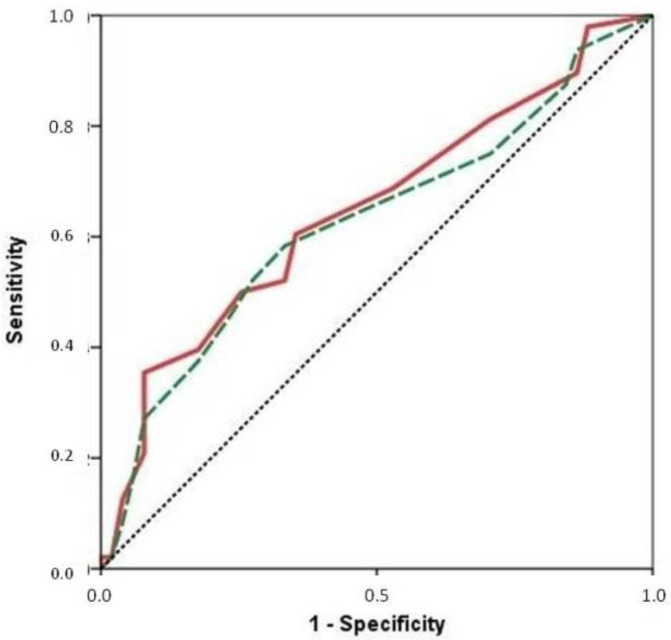
ROC curve showing the diagnostic performance of the SPPB (dashed green line) and the modified-SPPB (continuous red line) scale to detect frailty defined as FRAIL score > 3 in the older group of patients.

The SPPB scale was significantly related to frailty as measured by the FRAIL test in the young patients (6.59 ± 4.34 points vs. 9.88 ± 2.32 points; *p* = 0.007) and in the elderly patients (4.10 ± 3.00 points vs. 5.61 ± 3.51 points; *p* = 0.018). In the young patients, this scale showed an AUC of 0.72 (Supplementary Figure S3), and in the elderly patients, the AUC for frailty detection was 0.63 (FRAIL score > 3). If we add NT-proBNP to the SPPB scale in the older group by adding one point if the patients had an NT-proBNP lower than 4000 pg/mL, then the diagnostic performance of the SPPB-modified scale was slightly better (AUC: 0.65) (Figure 1).

## 4. Discussion

Frailty is a syndrome that has multiple definitions and a complex underlying pathophysiology. It is a reflection of biological rather than chronological age that contributes to the heterogeneity in clinical outcomes within the elderly patient population [11]. Frailty is particularly relevant in HF patients because it has been demonstrated that functional status, comorbidities, and cognitive function are associated with increased mortality risk in older patients with HF [12]. HF is a leading cause of hospitalization and is associated with poor prognosis in older patients [13]. In fact, frailty associated with HF has mostly been studied in the elderly, and evidence about its implication in young patients is lacking.

In addition, frailty scales consider comorbidities and physical parameters, and both are more frequent in elderly people than they are in younger people. This might result in lower scores on these scales in younger patients than in older patients. In this sense, the FRAIL scale, which was used to divide our sample into two age groups, is used to screen for frailty. It is a validated five-item questionnaire that asks questions about fatigue, resistance, ambulation, comorbidities, and weight loss. It predicts functional decline, mortality, and healthcare utilization [14]. Furthermore, the SPPB assesses the physical functional status in the elderly using balance, speed, and strength measurements. It has demonstrated its capability to predict dependency, institutionalization, hospitalization, and mortality [15].

On the other hand, frailty is sometimes caused by HF itself. Because of this, frailty regression has been demonstrated after a prescription for HF treatment [16] and after valvulopathy correction [17]. Biomarkers are objective measurements of the severity of cardiac disease, mainly HF, and are related to the clinical course of the disease. As such along with GFR, CRP, and hemoglobin, we also considered other biomarkers that are involved in cardiac damage, fibrosis, and stretch, such as troponin T ultrsensible, ST2, and NT-proBNP, respectively. For these reasons, we hypothesize that biomarkers might modulate the effect of chronological age on frailty scales scores.

In our sample of patients admitted to hospital for AHF, the prevalence of frailty measured by the FRAIL test was 33.7%, which is slightly lower than previous evidence [18]. The mean age was 73 ± 12 years old, but about half of patients were younger than 75 years old, similar to other studies [19]. This fact makes it especially important to set up new tools to improve the connection between frailty scores and HF results in both young and old patients.

In the younger group of patients, frailty was mainly determined by age and comorbidities. As we mentioned above, age is related to frailty in HF patients, which is probably due to a higher prevalence of comorbidities and functional disability [20]. The association between hypertension and diabetes with frailty has been studied in depth in previous research. The underlying mechanisms are diverse, but in a few words, it seems that some of the drugs that are used in these patients are involved in muscle weakness [21], and insulin resistance has a role in skeletal muscle function [22].

In the group of older patients, none of the baseline characteristics were related to frailty. This fact could be explained by a more homogenous distribution of comorbidities among older people. Furthermore, the age range in this group was narrower, and frailty might be more influenced by functional status and cognitive impairment than by age.

In younger patients experiencing heart failure, we did not find any association between the biomarkers and frailty, which was probably due to these young patients presenting with a shorter clinical course of HF, and it is likely that the disease has not yet affected physical performance. Cardiac cachexia and functional disability are phenomena that typically affect patients experienced end-stage HF [23]. This also explains the significant increase in NT-proBNP in the frail older patients in our sample. The best cut-off point for detecting frailty using NT-proBNP was 4000 pg/mL. Higher NT-proBNP cut-off values than those that are usually used for HF diagnosis could be more suitable for increasing the sensitivity to better discriminate frailty in these patients.

In the older group of patients, only NT-proBNP was related to frailty. The plasmatic levels of this biomarker increase with the HF stage and predict mortality and readmission among patients aged ≥65 years who have been hospitalized for HF [24]. Previous data have shown a relationship between NT-proBNP with muscle weakness [25] and functional disability [26]. In addition, BNP has been related to grip strength and gait speed [27]. The underling mechanisms that explain this connection have not been analyzed in deoth. First, BNP and NT-proBNP are secreted in response to cardiac stretch, which is increased in cases of muscle mass loss [28]. Second, the release of these biomarkers provokes energy dissipation and oxidative stress on muscular tissues [29]. Finally, BNP stimulates the excessive production of free fatty acids, which impair insulin sensitivity and muscle lipotoxicity [29].

In our study, the SPPB scale was significantly related to the frailty measured by the FRAIL test in both age groups. If we add NT-proBNP to SPPB scale by adding one point if the patient had a NT-proBNP lower than 4000 pg/mL, then the diagnostic performance of the SPPB-modified scale was slightly better. This fact might reflect that using biomarkers in addition to the usual geriatric scales could be useful for determining frailty more accurately in older HF inpatients. This does not mean that the multidimensional nature of frailty syndrome should be reduced to the measurements of some biomarkers, but they might complement geriatric assessment in HF patients.

The main strength of our study was that it analyzed the different performance of frailty scales in young and old patients admitted to the hospital for HF. Similar to us, Woo et al. showed a better relationship between frailty and HF severity in patients older than 75 years old with HF (in this case, only with preserved ejection fraction) than in younger patients [30]. They also connected NT-proBNP and high degrees of frailty, suggesting a possible role for this biomarker in the geriatric assessment of these patients. This association seems to be mediated by higher diastolic dysfunction in older frail patients, so, as mentioned above, HF itself might have a role in the prevalence of frailty.

Our article presents several limitations. First, we used a cross-sectional design in a relatively small sample, so we did not analyze variations in the biomarker levels or frailty scores. Second, we studied the biomarkers during admission for AHF, so the plasmatic levels might have been influenced by the acute severity of the episode. Third, some data such as the time from the HF diagnosis were not available. Fourth, the NT-proBNP assessment was performed at admission, and the geriatric evaluation was performed just before discharge; this might have an implication in clinical practice. Finally, most of the biomarkers are also considered biological markers of aging that could also be associated with the aging process independent of the presence of frailty [31,32]. Nevertheless, we believe that our study reflects the usefulness and feasibility of systematic assessment of the biomarkers in the geriatric evaluation of patients admitted for HF. 

## 5. Conclusions

In younger populations, frailty seems to be more determined by age and comorbidities; however, in the elderly population, it seems that the situation of myocardial and remodeling stress marked by NT-proBNP has an impact on frailty. The addition of biomarkers to traditional geriatric assessments could improve t frailty detection in the members of the elderly population who are admitted to the hospital for AHF.

## Figures and Tables

**Table 1:** Baseline characteristics in the group of young patients.

Variable	Frail Patients (*n*: 17; 16.67%)	Non-Frail Patients (*n*: 85; 83.33%)	*p*
Age (years old)	68 ± 5	62 ± 9	<0.001
Women	6 (35.3%)	25 (29.4%)	0.630
Hypertension	14 (82.4%)	48 (56.5%)	0.046
Diabetes	10 (58.8%)	21 (24.7%)	0.005
Atrial fibrillation	8 (47.1%)	31 (36.5%)	0.412
LVEF < 40%	7 (41.2%)	41 (48.2%)	0.595
ST2 (ng/mL)	128.11 ± 162.54	81.92 ± 83.01	0.300
Troponin T us (ng/L)	98.62 ± 246.36	73.66 ± 170.82	0.620
CRP (mg/L)	25.03 ± 21.21	21.72 ± 34.70	0.707
NT-proBNP (pg/mL)	8704.94 ± 8090.29	6028.73 ± 915.02	0.161
GFR (mL/min/1.73 m^2^)	56.56 ± 23.23	63.87 ± 23.74	0.142
Hemoglobin (g/dL)	12.93 ± 2.50	13.43 ± 2.32	0.417

LVEF: left ventricle ejection fraction. Us: ultrasensitive. CRP: C-reactive protein. GFR: glomerular filtration rate.

**Table 2:** Baseline characteristics in the group of old patients.

Variable	Frail Patients (*n*: 51; 51.51%)	Non-Frail Patients (*n*: 48; 48.48%)	*p*
Age (years old)	84 ± 5	82 ± 5	0.069
Women	22 (43.1%)	25 (52.1%)	0.373
Hypertension	44 (83.3%)	34 (70.8%)	0.060
Diabetes	18 (35.3%)	15 (31.2%)	0.670
Atrial fibrillation	31 (60.8%)	20 (41.7%)	0.057
LVEF < 40%	20 (39.2%)	17 (35.4%)	0.696
ST2 (ng/mL)	108.17 ± 84.16	85.49 ± 72.69	0.180
Troponin T us (ng/L)	149.55 ± 401.09	130.31 ± 245.94	0.776
CRP (mg/L)	26.30 ± 49.44	22.15 ± 35.07	0.636
NT-proBNP (pg/mL)	12,297.61 ± 13,710.20	7709.40 ± 8374.26	0.046
GFR (mL/min/1.73 m^2^)	46.24 ± 18.85	51.84 ± 17.23	0.127
Hemoglobin (g/dL)	12.74 ± 2.09	12.46 ± 1.85	0.480

LVEF: left ventricle ejection fraction. Us: ultrasensitive. CRP: C-reactive protein. GFR: glomerular filtration rate.

## Supplementary Materials

The following are available online at https://www.mdpi.com/article/10.3390/biom12020245/s1, Figure S1: SPPB scale, Figure S2: FRAIL scale, Figure S3: ROC-curve showing the diagnostic performance of the SPPB scale to detect frailty defined as FRAIL score > 3 in the younger group of patients, Table S1: HF treatment before admission.
