# Supplementary material for: Differences According to Age in the Diagnostic Performance of Cardiac Biomarkers to Predict Frailty in Patients with Acute Heart Failure
Source: Biomolecules. 2022 Feb 2;12(2):245. doi: 10.3390/biom12020245 (PMC8961634; doi:10.3390/biom12020245)
Supplement: Supplementary file 1 [file biomolecules-12-00245-s001.zip › biomolecules-1585288-supplementary.pdf]

Supplementary material

Figure S1. SPPB scale

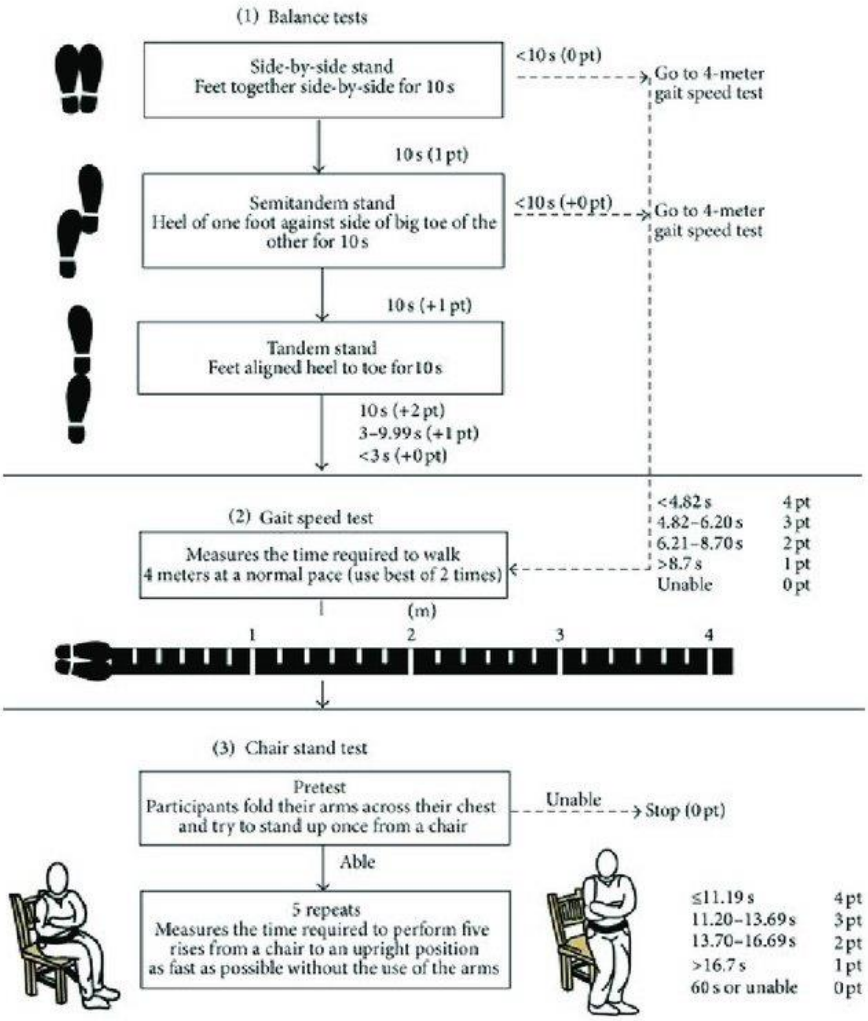

Mugueta-Aguinaga, I.; Garcia-Zapirain, B. FRED: Exergame to Prevent Dependence and Functional Deterioration Associated with Ageing. A Pilot Three-Week Randomized Controlled Clinical Trial. Int. J. Environ. Res. Public. Health 2017, 14, E1439, doi:10.3390/ijerph14121439.

Figure S2. FRAIL scale

The FRAIL Scale

Item	Scale
Fatigue	Tired all or most of the time during the past four weeks (No/Yes)
Resistance	Difficulty walking up 10 steps without resting or aids (No/Yes)
Ambulation	Difficulty walking several hundred yards alone without aid (500-600 meters) (No/Yes)
Illnesses	5 or more illnesses (No/Yes)
Loss of weight	Weight loss >5% within the past moth (No/Yes)

Frail Scale scores range from 0-5, one point for each component, 0=best to 5=worst

Robust = 0 points

Pre-Frail = 0-1 points

Frail = 3-5 points

Woo, J.; Yang, X.; Tin Lui, L.; Li, Q.; Fai Cheng, K.; Fan, Y.; Yau, F.; Lee, A.P.W.; Lee, J.S.W.; Fung, E. Utility of the FRAIL Questionnaire in Detecting Heart Failure with Preserved Ejection Fraction. J Nutr Health Aging 2019, 23, 373-377, doi:10.1007/s12603-019-1158-1.

Figure S3. ROC-curve showing the diagnostic performance of the SPPB scale to detect frailty defined as FRAIL score > 3 in the younger group of patients

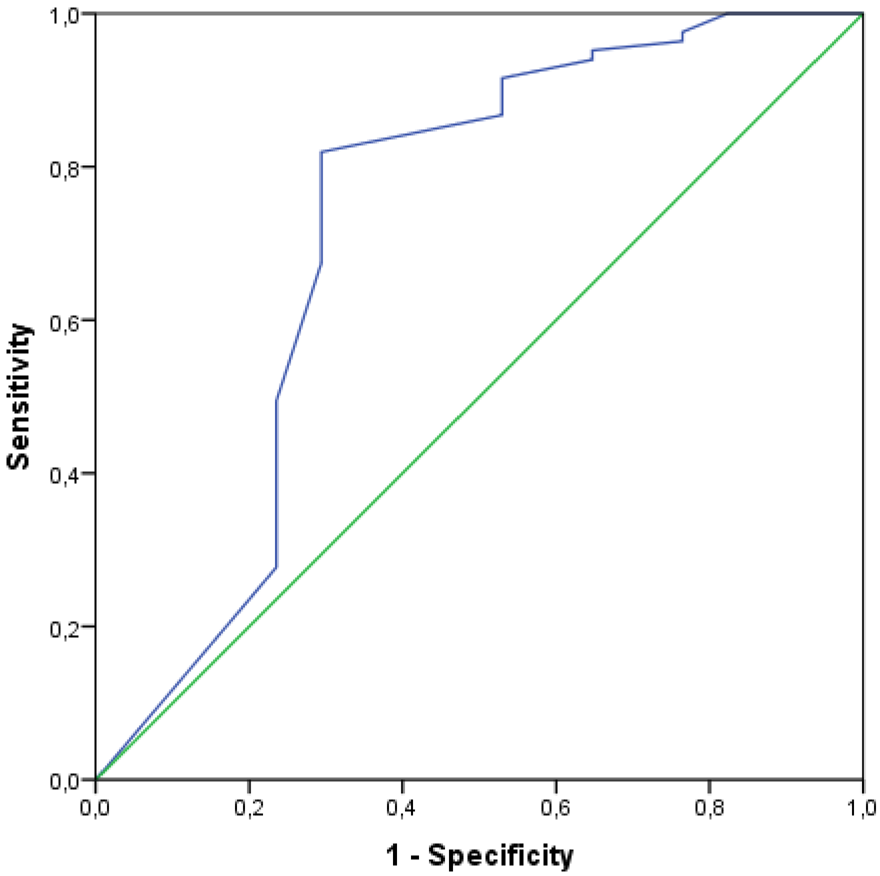

Table S1. HF treatment before admission

**Medication**	**N**	**%**
Angiotensin-converting-enzyme inhibitors or Angiotensin receptor blockers	84	41.6
Sacubitril-valsartan	23	11.4
Aldosterone antagonists	27	13.4
Betablockers	101	50.0
Sodium glucose co-transporter 2 inhibitors	19	9.4
Diuretics	110	54.4
